# Abundance of Plant-Associated *Gammaproteobacteria* Correlates with Immunostimulatory Activity of *Angelica sinensis*

**DOI:** 10.3390/medicines6020062

**Published:** 2019-05-31

**Authors:** Kriti Kalpana, Diego Montenegro, Giovanna Romero, Ximena Peralta, Betul Akgol Oksuz, Adriana Heguy, Moriya Tsuji, Akira Kawamura

**Affiliations:** 1 Department of Chemistry, Hunter College of CUNY, New York, NY 10065, USA; kritikalpana@gmail.com (K.K.); diego.montenegro@gmail.com (D.M.); gioromero25@gmail.com (G.R.); Ximenaperalta613@gmail.com (X.P.); 2 Biochemistry Ph.D. Program, The Graduate Center of CUNY, New York, NY 10016, USA; 3 Genome Technology Center, NYU Langone Medical Center, New York, NY 10016, USA; Betul.AkgolOksuz@umassmed.edu (B.A.O.); Adriana.Heguy@nyulangone.org (A.H.); 4 HIV and Malaria Vaccine Program, Aaron Diamond AIDS Research Center, Affiliate of The Rockefeller University, New York, NY 10016, USA; mtsuji@adarc.org; 5 Chemistry Ph.D. Program, The Graduate Center of CUNY, New York, NY 10016, USA

**Keywords:** microbe-associated molecular pattern (MAMP), lipopolysaccharide (LPS), *Angelica sinensis*, Dong quai, Shi-Quan-Da-Bu-Tang, Juzen-taiho-to

## Abstract

**Background:***Angelica sinensis* is a medicinal plant known for a variety of biological effects, including its ability to stimulate innate immune cells in humans. Recent studies indicate that the immunostimulatory activity of *A. sinensis* arises from microbe-associated molecular patterns (MAMPs) of plant-associated bacteria. However, it is unknown which bacterial taxa in *A. sinensis* are responsible for the production of immunostimulatory MAMPs. **Methods:** Samples of *A. sinensis* were subjected to a cell-based assay to detect monocyte-stimulation and 16S ribosomal RNA amplicon sequencing, which revealed their immunostimulatory activity and microbial communities. The resulting data were analyzed by Linear discriminant analysis effect size (LEfSe), an online biostatistical tool for metagenomic biomarker discovery, to identify the bacterial taxonomical features correlated with the immunostimulatory activity. **Results:** A series of bacterial taxa under *Gammaproteobacteria* correlated positively with the immunostimulatory activity, whereas several Gram-positive taxa and *Betaproteobacteria* correlated negatively with the activity. **Conclusions:** The identified bacterial taxa set a new stage to characterize immunostimulatory MAMPs in plants.

## 1. Introduction

Plants harbor numerous and diverse microbes. Plant-associated microbes play vital roles in the health of plants by producing nutrients, degrading wastes, and protecting their hosts from pathogens [1]. However, plants may not be the only beneficiaries of these microbes. Humans consume vegetables, fruits, and herbs, with which plant-associated microbes are also ingested. Although ingested microbes might not survive the passage through the stomach acid, their metabolites can remain intact and exhibit biological effects. Recent studies suggest that microbe-associated molecular patterns (MAMPs) of plant-associated bacteria modulate the human immune system. In a study on *Echinacea*, an immune-boosting medicinal herb, Pasco and co-workers revealed that MAMPs are responsible for the ability of *Echinacea* to stimulate innate immune cells, such as macrophages [2,3]. They also found a correlation between the total bacterial load in *Echinacea* and macrophage activity [4]. Independently, Cech and co-workers found that *Echinacea*, when grown under a germ-free condition, did not stimulate innate immune cells [5], which further underscored the importance of MAMPs for the immunostimulatory activity of *Echinacea*. Recently, our group found that lipopolysaccharides (LPSs), prototypical MAMPs of Gram-negative bacteria, are the key immunostimulants in Juzen-taiho-to (JTT) [6], which is an immune-boosting herbal formulation in East Asia; JTT is clinically used in Japan to boost the immunological functions of patients with cancer [7,8,9], hepatitis C [10], otitis [11], and anemia [12]. These findings support the emerging notion that MAMPs of environmental bacteria play important roles in the development and maintenance of our immune system [13,14].

Although the MAMPs of human pathogens, also known as pathogen-associated molecular patterns (PAMPs), have long been the subject of biomedical research, little is currently known about the MAMPs of phytobacteria. A few known ones, including the LPSs of *Rhizobium etli*, elicit little or no immunological responses [15]. As such, it had been generally assumed that phytobacterial MAMPs are structurally too diverged from PAMPs to exhibit immunological effects. The recent findings of immunostimulatory MAMPs in *Echinacea* and JTT, however, suggest that phytobacterial MAMPs can serve as an untapped source of immunostimulatory agents. Thus, it is important to start characterizing their chemical structures as well as immunological effects.

It is, however, not a trivial task to characterize immunostimulatory MAMPs in plants. Each plant contains numerous and diverse bacteria. Each bacterium produces multiple MAMPs. As such, plants contain enormously complex mixtures of MAMPs from hundreds or possibly thousands of different bacterial species. Furthermore, the amounts of individual MAMPs in plants are minuscule compared to phytochemicals produced by plants. The staggering chemical complexity as well as minuscule quantities defy the classical approach of natural products chemistry; that is, purification of active compounds followed by structural and biological characterization. Even if a few MAMPs could be purified, it would not help in terms of gaining a holistic understanding of the immunostimulatory activity of medicinal plants.

An alternative approach is to harness the enormous screening power of amplicon sequencing. The genetic data of bacteria, such as 16S ribosomal RNA (16S rRNA), can be amplified in the presence of plant DNA. Amplified bacterial genes can then be sequenced to obtain comprehensive profiles of bacterial communities in plants, which reflect the diversity of MAMPs. The resulting data can be subjected to bioinformatics analyses to find the taxa correlated with the immunostimulatory activity of plants. Once the bacterial taxa of interest are identified, subsequent studies can focus on their MAMPs to understand their effects on the human immune system. Although various Gram-negative taxa have been detected in immune-boosting plants previously [6,16], it remains to be determined which bacterial taxa are correlated with the immunostimulatory activity.

Here, we present the phytobacterial taxa that correlate with the in vitro immunostimulatory activity of *Angelica sinensis*, which is a key component herb in JTT [6]. In this study, the immunostimulatory activity of *A. sinensis* was first examined by a cell-based assay using THP-1 monocytes, which revealed a substantial sample-to-sample variation. In addition, the microbial community structure of *A. sinensis* was determined by 16S rRNA amplicon sequencing, which also revealed a large sample-to-sample variation. The immunological data and the 16S profiles were then subjected to Linear discriminant analysis effect size (LEfSe) [17]. The LEfSe analysis revealed several bacterial taxa strongly correlated with the immunostimulatory activity of *A. sinensis*. The identified taxa provide a new basis to start characterizing immunostimulatory MAMPs in plants.

## 2. Materials and Methods

### 2.1. Materials

The solvents for extraction and purification were HPLC grade and were purchased from VWR (Radnor, PA, USA) and Fisher Scientific (Waltham, MA, USA). Four different batches (dried roots) of *A. sinensis* (Dong quai), namely, AS1, AS2, AS3, and AS4, were obtained from local Chinese Pharmacies in New York, NY, USA. Unless specified otherwise, all other chemicals and reagents were obtained from Fisher Scientific and VWR and used without further purification.

### 2.2. Preparation of A. sinensis Extracts for the Cell-Based Assay

Dried pieces of *A. sinensis* (10 g) were extracted with boiling water (100 mL) for one hour. Insoluble materials were removed by vacuum filtration. The filtrate was dried and reconstituted in DMSO to obtain a stock solution of *A. sinensis* extract (1 µg/µL) for cell treatments.

### 2.3. Cell Culture

THP-1 monocytes were purchased from ATCC and cultured in RPMI-1640 medium containing 25 mM HEPES and L-glutamine (HyClone) and supplemented with 10% (*v*/*v*) fetal bovine serum (Fisher), 0.005 mM β-mercaptoethanol (Fisher), and a mixture of 1% (*v*/*v*) penicillin, streptomycin, and amphotericin B (VWR). The cells were maintained at 37 °C in a 5% CO_2_ incubator.

### 2.4. Cell Treatment and RNA Purification

THP-1 cells were plated at a concentration of 500,000 cells/2 mL in each well of a 12-well plate. On the following day, cells were treated with *A. sinensis* extracts to achieve a final concentration of 5 µg/mL, positive control (JTT at 100 µg/mL and LPS at 0.1 ng/mL), or DMSO as the vehicle control, for 4 h in a 37 °C incubator and 5% CO_2_. After 4 h, cells were transferred to a 15 mL Falcon tube and centrifuged at 1300× rpm for 5 min at room temperature. The medium was aspirated, and the pellet was subjected to RNA purification using an E.Z.N.A. Total RNA Kit I (Omega Bio-Tek, Norcross, GA, USA). The resulting RNA samples were quantified by UV_260_ absorption, which was measured by a NanoDrop 1000 Spectrophotometer (Thermo Fisher, Waltham, MA, USA). Samples showing the 260 nm/280 nm ratios between 1.8 and 2.2 were used for cDNA synthesis.

### 2.5. Reverse Transcription and Quantitative Polymerase Chain Reaction

The purified RNA samples were used to prepare cDNA by reverse transcription (RT). For each RT reaction, 1 µL of a 10 mM dNTP Mix (Promega) and 2 µL of random primers (0.3 µg/µL, Invitrogen) were added to 13 µL of RNA samples (200 ng/µL). The mixture was placed in a PCR thermocycler for 3 min at 70 °C and then cooled to 4 °C for 1 min. To each of the reaction tubes, 4 µL of 5× RT buffer and 0.5 µL of 200 U/µL M-MLV Reverse Transcriptase (Promega) were added. The resulting mixture was incubated at 42 °C for 1 h and then heated to 95 °C for 10 min. Finally, the mixture was incubated at 37 °C for 20 min. The synthesized cDNA samples were diluted with diethyl pyrocarbonate (DEPC) treated water to 0.025 µg/µL for quantitative polymerase chain reaction (qPCR) analysis. The expression level of intercellular adhesion molecule 1 (ICAM-1) in each sample was determined by Taqman^®^ Gene Expression assays (Applied Biosystems) using a 7500 Real-Time PCR system (Applied Biosystems, Beverly, MA, USA), in which GAPDH was used as the endogenous control. For each sample, triplicate experiments were carried out. The relative gene expression or fold-change (relative quantitation) was calculated by the ΔΔC_T_ method.

### 2.6. DNA Extraction from A. sinensis for 16S rRNA Amplicon Sequencing

For each DNA extraction, dried pieces of *A. sinensis* sample (ca. 0.25 g) were ground to powder containing 2–5 mm particles using a coffee bean grinder. The resulting powder was subjected to DNA isolation using the MoBio PowerLyzer^®^ PowerSoil^®^ DNA Isolation kit. Purified DNA samples were quantified with Nanodrop^®^ to obtain approximately 1.2–1.6 µg of DNA per sample. DNA extraction was carried out in three independent replicates for each sample of *A. sinensis*.

### 2.7. 16S rRNA Amplicon Sequencing and Data Analysis

DNA samples, which contained DNA from both microbes and plants, were subjected to 16S rRNA amplicon sequencing. The study used the primer pair (799f/1114r) that can selectively amplify bacterial 16S rRNA genes in the presence of chloroplast and mitochondrial DNA from *A. sinensis* samples. Although another primer pair (799f/1392r) is more commonly used to amplify bacterial 16S rRNA from plant samples, our preliminary PCR analysis of *A. sinensis* with the 799f/1392r primer pair gave an unexpectedly large PCR amplicon as well as the expected product. On the other hand, from the 799f/1114r primer pair, which has been used to amplify 16S rRNA from Chinese cabbage [18], a single PCR product was obtained. Thus, the current study used the 799f/1114r primer pair. The study followed the 16S Illumina sequencing protocol as described by Caporaso et al. [19], which gave 2 × 300 Illumina MiSeq reads. After quality analysis of the raw reads (Illumina BaseSpace, San Diego, CA, USA) and fastq raw sequence file generation, the data set was subjected to analysis with Quantitative Insights Into Microbial Ecology (QIIME) version 1.8 [20] on Amazon EC2 Cloud Computing Clusters. Barcodes were extracted from the raw reads, and the headers of forward reads were matched with those of corresponding reverse reads. Paired-ends were joined (a minimum overlap of 10 bases; a percent maximum difference of 20%). The joined reads were then demultiplexed with a Phred quality score threshold of Q20 or higher. The high quality reads were subjected to a closed-reference operational taxonomic unit (OTU)-picking process using the BLAST algorithm aligning reads against the GREENGENES (gg_otus-13_8-release) 97_otu database, followed by phylogenetic diversity analysis with a sampling depth of 5000 sequences per sample.

The potential metagenomic biomarkers were identified by LEfSe [17] on the Galaxy platform [21]. The OTU table (Supplementary Table S1) from the QIIME analysis was subjected to LEfSe. LEfSe detected OTU-features with significant differences between the High and Low activity groups by class and sub-class comparison analyses using the Kruskal–Wallis sum-rank test (Alpha value threshold: 0.05) and the Wilcoxon rank-sum test (Alpha value threshold: 0.05), respectively. Finally, the effect size of each differentially abundant feature was estimated by Linear Discriminant Analysis (LDA) to obtain microbial taxa with LDA values of 4.5 or higher, which is much more stringent than the default threshold of LEfSe (LDA = 2).

## 3. Results

**Figure 1:**
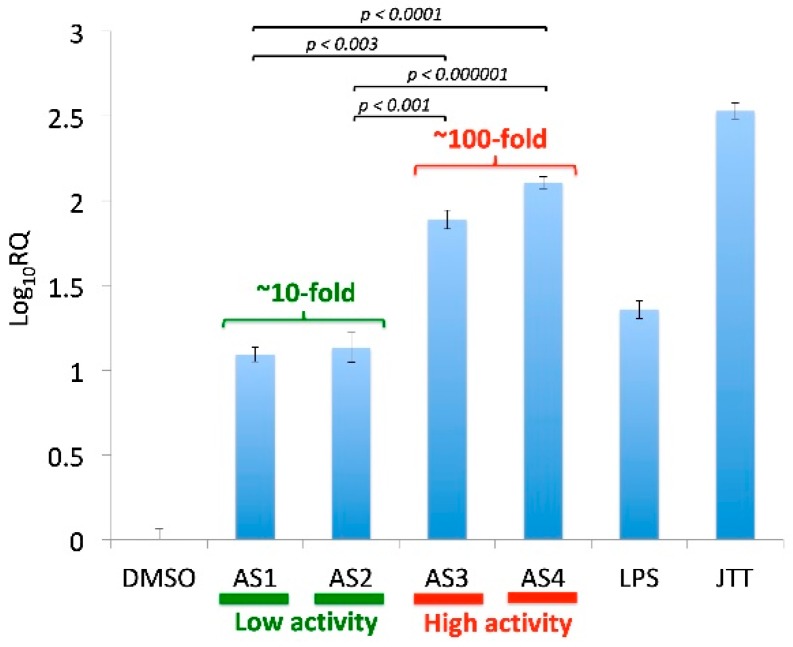
Immunostimulatory activity of four *Angelica sinensis* samples. The activity was measured by qRT-PCR of ICAM-1 in THP-1 monocytes. Cells were treated with samples for four hours; JTT (Juzen-taiho-to, 100 μg/mL, positive control); lipopolysaccharide (LPS) (*Escherichia coli* LPS, 0.1 ng/mL, positive control), *A. sinensis* samples (5 μg/mL); DMSO (vehicle control). Each sample was analyzed at least in triplicate (*n* = 3). RQ (Relative Quantitation): fold change. AS3 and AS4 showed much higher activities (77-fold and 128-fold, respectively) than AS1 and AS2 (both ~12-fold).

Four different samples of *A. sinensis* (AS1, AS2, AS3, and AS4) were examined in the current study. The immunostimulatory activity was determined by the messenger RNA (mRNA) induction of ICAM-1 in human THP-1 monocytes [22]. In this assay, induction of ICAM-1 mRNA in THP-1 cells was determined based on the comparison with the DMSO control. This assay indicated that the four samples (AS1, AS2, AS3, AS4) could be grouped into two classes, namely, (1) Low activity (AS1, AS2) and (2) High activity (AS3, AS4) (Figure 1). While the low-activity samples (AS1, AS2) caused about 10-fold induction of ICAM-1 in THP-1 monocytes, the high-activity samples (AS3, AS4) exhibited around 100-fold induction. The two classes (Low and High) of *A. sinensis* samples set the stage to look for differentially abundant bacterial taxa.

**Figure 2:**
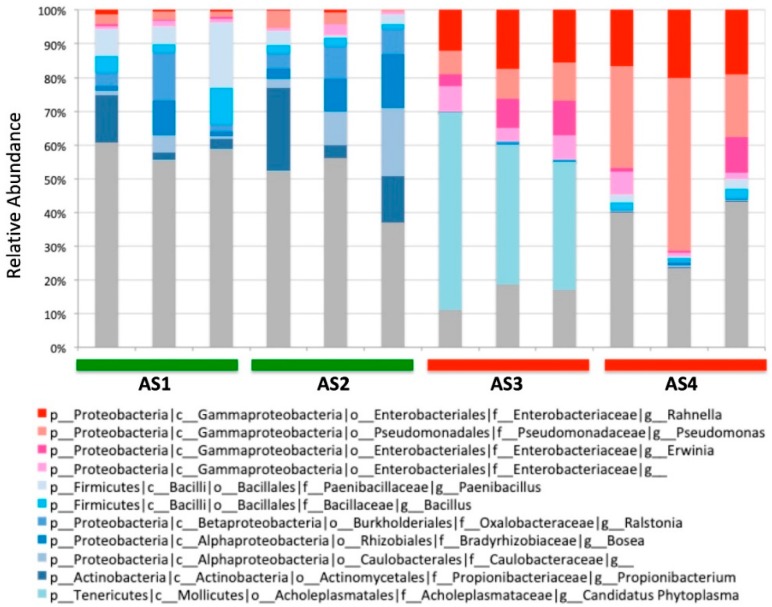
Microbial communities of four *A. sinensis* samples at the genus level. The most abundant genera are shown. The genera in the class *Gammaproteobacteria* are colored in reddish hues. Other abundant genera are shown in bluish colors. Three independent replicates were made for each sample. p: phylum; c: class; o: order; f: family; g: genus.

16S rRNA amplicon sequencing was carried out with Illumina MiSeq using a primer pair (799f/1114r) that minimizes the co-amplification of chloroplast and mitochondrial DNA in plants [23]. For each *A. sinensis* sample, three independent replicates were made to account for the possible variability within the same sample. As such, a total of 12 DNA samples were prepared and subjected to the amplicon sequencing. The sequencing revealed diverse bacterial communities with a total of 859 different genera (Figure 2). The sequencing also showed that each sample harbors a distinctly different bacterial community. Even the replicates of the same sample showed some variability, suggesting that each sample consists of a heterogeneous mixture of the dried pieces of *A. sinensis*. It appeared that several genera in the class of *Gammaproteobacteria* were associated with the High-activity group, such as *Pseudomonas* and *Rahnella*, although more rigorous statistical analysis was needed to examine the correlation.

In order to identify the taxonomic features correlated with the two classes of immunostimulatory activity (Low and High), the bacterial profiles were analyzed by LEfSe [17], which examines not only the statistical significance of differentially distributed features but also evaluates biological consistency and effect size. We used LEfSe for the current study because, in addition to its statistical rigor, LEfSe is an online tool on the Galaxy platform [24], which is available to any researcher interested in similar metagenomic biomarker analyses of medicinal plants.

**Figure 3:**
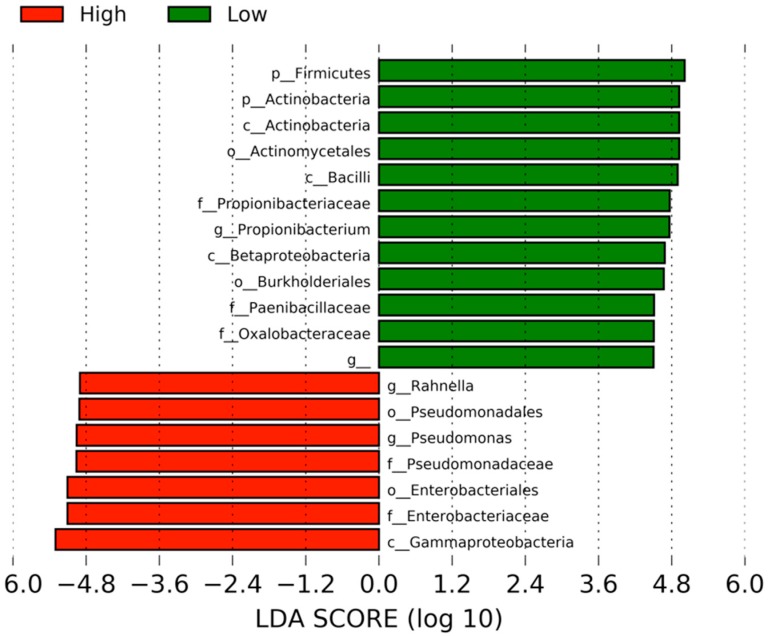
Differentially abundant clades between the high- and low-activity samples of *A. sinensis*. The clades correlated with the High monocyte activity are shown in red, whereas those correlated with the Low activity are in green. The default linear discriminant analysis (LDA) threshold in Linear discriminant analysis effect size (LEfSe) is 2.0. The higher threshold makes the analyses more stringent.

LEfSe identified 19 differentially abundant clades with a linear discriminant analysis (LDA) score of 4.5 or above (Figure 3). Seven clades were abundant in the High activity group: namely, one class (*Gammaproteobacteria*), two orders (*Enterobacteriales* and *Pseudomonadales*), two families (*Enterobacteriaceae* and *Pseudomonadaceae*), and two genera (*Rahnella* and *Pseudomonas*). On the other hand, the abundant clades in the Low-activity group were two phyla (*Firmicutes* and *Actinobacteria*), three classes (*Bacilli*, *Actinobacteria*, and *Betaproteobacteria*), two orders (*Actinomycetales* and *Burkholderiales*), three families (*Propionibacteriaceae*, *Paenibacillaceae*, and *Oxalobacteraceae*) and two genera (*Propionibacterium* and an undefined genus in the *Caulobacteraceae* family).

**Figure 4:**
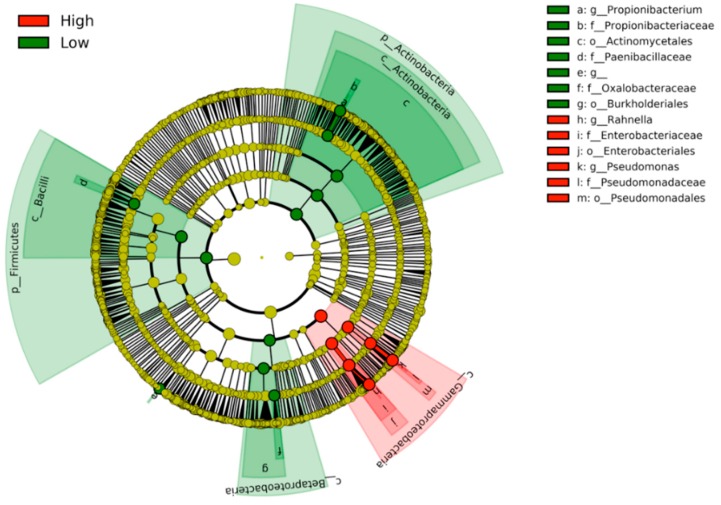
Cladogram showing the distribution of the clades correlated with the High monocyte activity (red) and the Low activity (green). The High-activity clades are clustered within the class *Gammaproteobacteria*.

When the discriminative clades were visualized by the cladogram, the High- and Low-activity groups were clearly separated (Figure 4). The clades correlated with the High-activity group were all localized within *Gammaproteobacteria*, which is a class of Gram-negative bacteria. On the other hand, the Low-activity clades were seen in two phyla of Gram-positive bacteria (*Firmicutes* and *Actinobacteria*) and a class of Gram-negative bacteria (*Betaproteobacteria*).

**Figure 5:**
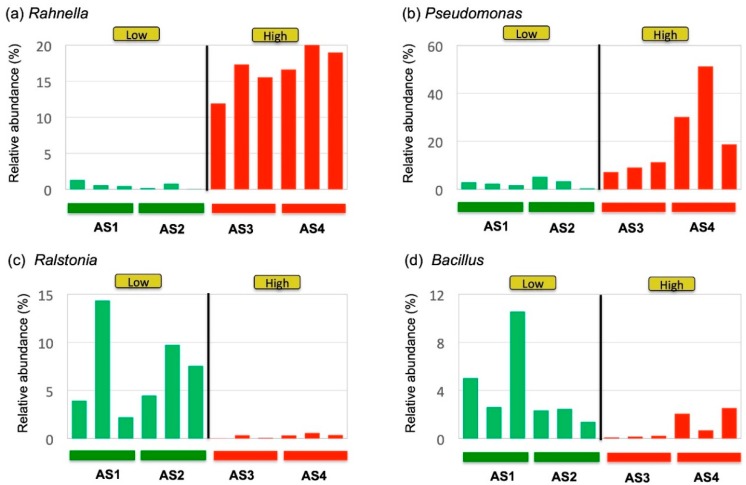
Plots of bacterial genera correlated with the in vitro immunostimulatory activity, (**a**) *Rahnella* and (**b**) *Pseudomonas*, and Low activity, (**c**) *Ralstonia* and (**d**) *Bacillus*. Three independent replicates were made for each sample to show the variability of microbial community within the same sample. The genera *Rahnella* and *Pseudomonas* were abundant in High-activity samples (AS3 and AS4), whereas *Ralstonia* and *Bacillus* were abundant in Low-activity samples (AS1 and AS2).

Closer examination of the observed clades at the genus level revealed that some genera were more consistently associated with the activity than others (Figure 5). For example, the genus *Rahnella* was consistently higher in the High-activity samples than in the Low-activity group (Figure 5a). On the other hand, the abundance of the genus *Pseudomonas* varied within the High-activity samples, although it was still significantly higher in the High-activity samples (Figure 5b). Likewise, the abundance of the Gram-positive genus *Ralstonia* was consistently higher in the Low-activity samples (Figure 5c), whereas the abundance of the Gram-positive genus *Bacillus* varied substantially from sample to sample (Figure 5d).

## 4. Discussion

The advent of powerful sequencing tools has transformed the way we characterize plant-associated microbes. Microbial communities of both phyllosphere and rhizosphere have been characterized for various plants, including *Arabidopsis* [25,26], maize [27], basil [28], ginger [29], lettuce [30], and Korean ginseng [31]. These studies provided important new insights into plant–microbe interactions, which can be translated to agricultural applications, such as development of biocontrol agents and pathogen detection. However, few studies have examined the potential link between plant-associated microbes and therapeutic effects of medicinal plants. To our knowledge, the current study is the first to examine the correlation between the plant-associated microbes and the immunomodulatory activity of *A. sinensis*, which is a key herbal component in JTT [6]. Admittedly, the sample size of the current study is small, which underscores the importance of further studies to verify our findings. Yet, several exciting new hypotheses emerged from the current study as outlined below.

First, the current study revealed the correlation between the abundance of *Gammaproteobacteria* in *A. sinensis* and high monocyte activity. The finding suggests that MAMPs of some plant-associated *Gammaproteobacteria* might elicit strong immunological responses. The class *Gammaproteobacteria* includes many human pathogens, such as *Salmonella*, *Yersinia*, and *Vibrio* [32]. Although plant-associated *Gammaproteobacteria* have diverged from human pathogens, their MAMPs might still retain some structural features of PAMPs. Among the bacteria in *Gammaproteobacteria*, several genera correlated strongly with the high monocyte activity: namely, *Rahnella* and *Pseudomonas*. Identification of these genera sets the stage to further characterize their MAMPs, such as LPSs, to see if they are responsible for the immunostimulatory activity of *A. sinensis*. Although there have been studies on the polysaccharide moieties of *Rahnella* LPSs [33,34,35,36,37], their lipid A moieties, which are responsible for the activation of monocytes, have not been characterized.

Second, the current study also opens a possibility to use the observed taxa as biomarkers to predict the immunostimulatory activity of *A. sinensis* and possibly other medicinal plants. The taxa of interest, such as *Gammaproteobacteria*, can be quickly quantified by qPCR. As such, it may become possible to standardize the immunostimulatory activity of *A. sinensis* samples by simple qPCR assays. Simple assays to standardize medicinal plants are important because they could mitigate sample-to-sample variability, which has long been a major issue in herbal medicine [38,39]. Although chemical markers have been developed for the quality control of some medicinal plants [40,41], many herbs, including *A. sinensis* [42], lack effective methods to standardize their quality. Thus, the current study opens an intriguing possibility to use plant-associated bacteria as “biosensors” to control the quality and reproducibility of medicinal plants.

Finally, our study provides a new mechanistic insight into the immunostimulatory effects of JTT and *A. sinensis* (Dong quai). Both JTT and *A. sinensis* are known to stimulate innate immune cells [22,43]. In particular, it is well-recognized that JTT exhibits “LPS-like” immunological effects [44,45] as evidenced by very similar gene expression profiles of JTT and LPS [22,46]. The current study further corroborates the emerging notion that humans may be benefiting from plant-associated bacteria through the practice of herbal medicine.

In conclusion, the current study revealed a correlation between plant-associated *Gammaproteobacteria* and in vitro immunostimulatory activity of *A. sinensis.* The finding sets the stage for further studies to characterize the potential roles of plant-associated bacteria in *A. sinensis* and other immunostimulatory medicinal plants.

## Supplementary Materials

The following are available online at https://www.mdpi.com/2305-6320/6/2/62/s1, Table S1: OTU Table (Level 6: the genus level) from the QIIME 1.8.0 analysis.
